# Septic shock from descending necrotizing mediastinitis – combined treatment with IgM-enriched immunoglobulin preparation and direct polymyxin B hemoperfusion: a case report

**DOI:** 10.1186/s13256-018-1611-5

**Published:** 2018-03-03

**Authors:** Vincenzo Pota, Maria Beatrice Passavanti, Pasquale Sansone, Maria Caterina Pace, Filomena Peluso, Alfonso Fiorelli, Caterina Aurilio

**Affiliations:** 1 Department of Women, Infant and Surgical and Specialist Surgery, University of Campania “L. Vanvitelli”, Piazza Miraglia 2, 80138 Naples, Italy; 2 Thoracic Surgery Unit, University of Campania “L. Vanvitelli”, Naples, Italy

**Keywords:** Septic shock, IgM-enriched immunoglobulin preparation, Polymyxin B hemoperfusion, Mediastinitis

## Abstract

**Background:**

Descending necrotizing mediastinitis is a common and progressive polymicrobial infection involving the neck and chest with a high death rate (10 to 40%). From a microbiological point of view, descending necrotizing mediastinitis is sustained by Gram-positive bacteria (43–62%), anaerobes (46–78%), and, rarely, Gram-negative bacteria. Data collected during the Antibiotic Resistance-Istituto Superiore di Sanità project confirmed that Italy is positioned among the countries with the highest levels of resistance in most pathogenic species under surveillance. In particular, 32.9% of *Klebsiella pneumoniae* isolates were resistant to carbapenem, 33.6% of *Staphylococcus aureus* to methicillin, and 28.7% and 43.9% of *Escherichia coli* isolates to third-generation cephalosporins and fluoroquinolones, respectively.

**Case presentation:**

We describe the case of a 38-year-old white man with septic shock due to descending necrotizing mediastinitis sustained by multidrug-resistant Gram-negative and Gram-positive bacteria treated after surgery with an IgM-enriched immunoglobulin preparation and polymyxin B hemoperfusion therapy.

**Conclusion:**

Despite the contrasting data on the use of immunoglobulins and polymyxin B hemoperfusion in septic shock and the lack of literature in cases of acute mediastinitis caused by both Gram-negative and Gram-positive multidrug-resistant bacteria, we obtained an improvement in clinical conditions and the survival of our patient, against all odds.

## Background

Acute mediastinitis (AM), because of its high mortality rate, is one of the most dangerous forms of infection in the human organism. It is a severe acute inflammation of the connective tissues located in the middle thoracic cavity. Descending necrotizing mediastinitis (DNM) represents 20% of cases of AM [1, 2]. DNM is a common and progressive polymicrobial infection involving the neck and chest and it is associated with a high death rate (10 to 40%).

There are no guidelines or published articles with high level of evidence (above level III) on the treatment of DNM. Currently, the best evidence is available from four meta-analyses [3–6] of published case series of DNM, covering the period from 1960 to 2008, and a review of cervical necrotizing fasciitis and DNM [7]. The primary treatment of DNM is surgical eradication of the pharyngeal or odontogenic focus and a concomitant extensive drainage of the neck and the mediastinum. The thoracic surgical treatment could be a median sternotomy, or standard right posterolateral thoracotomy, or anterolateral thoracotomy, or subxiphoid approach, or clamshell incision, or video-assisted mediastinoscopy (VAM) drainage, and/or video-assisted thoracic surgery (VATS) drainage. Unfortunately, the literature did not offer a consensus on the optimal treatment modalities [6]. Surely, the surgical treatment has to be combined with an intravenously administered wide-spectrum antibiotic therapy and other intensive care treatment. From a microbiological point of view, DMD is sustained by Gram-positive bacteria (43–62%), anaerobes (46–78%), and, rarely, Gram-negative bacteria.

We describe a case of AM caused by an odontogenic infection due to methicillin-resistant *Enterococcus raffinosus*, carbapenem-resistant *Acinetobacter baumannii* (CRAB), and carbapenem-resistant *Klebsiella pneumoniae* (CR-Kp) unresponsive to conventional surgical and medical therapy complicated with high levels of procalcitonin (PCT), endotoxin, and septic shock.

## Case presentation

A 38-year-old white man, 100 kg weight, with a diagnosis of DNM, was transferred to the intensive care of University of Campania “L. Vanvitelli” because of the necessity of a chest surgery-dedicated intensive care unit (ICU). He came from an ICU of a peripheral hospital with the incorrect diagnosis of pneumonia, based on a chest X-ray. He was treated with tazobactam (2 g/day)/piperacillin (16 g/day) and meropenem (6 g/day) for approximately 10 days and percutaneous tracheostomy. When he arrived at our ICU he presented respiratory failure with the necessity of mechanical ventilation with partial pressure of oxygen in arterial blood (PaO_2_)/fraction of inspired oxygen (FiO_2_) < 90. He was in septic shock with severe hypotension with necessity of norepinephrine > 0.3 μg/kg per minute. His mean arterial pressure (MAP) was 50 mmHg, heart rate 130 beats per minute (bpm), and body temperature 40 °C.

His medical history was: amoxycillin (2 g/day) had been used to treat his severe toothache for 2 weeks and then he presented to the emergency room of a peripheral hospital with dyspnea. He was obese (body mass index > 39) but was not affected by any other comorbidities. He was single and he worked as a truck driver. He did not smoke tobacco or drink alcohol and he did not have any other risk factor for mediastinitis.

**Fig. 1:**
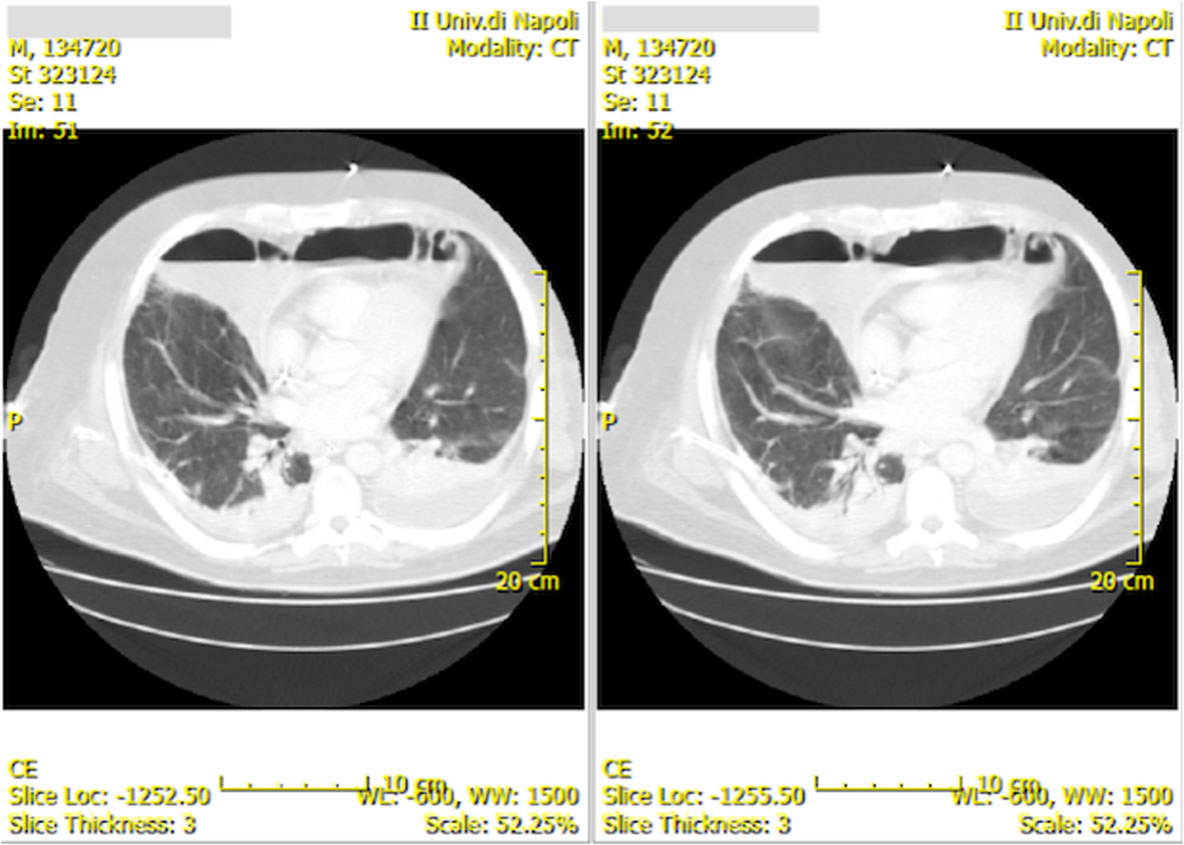
Cervical computed tomography scan

**Fig. 2:**
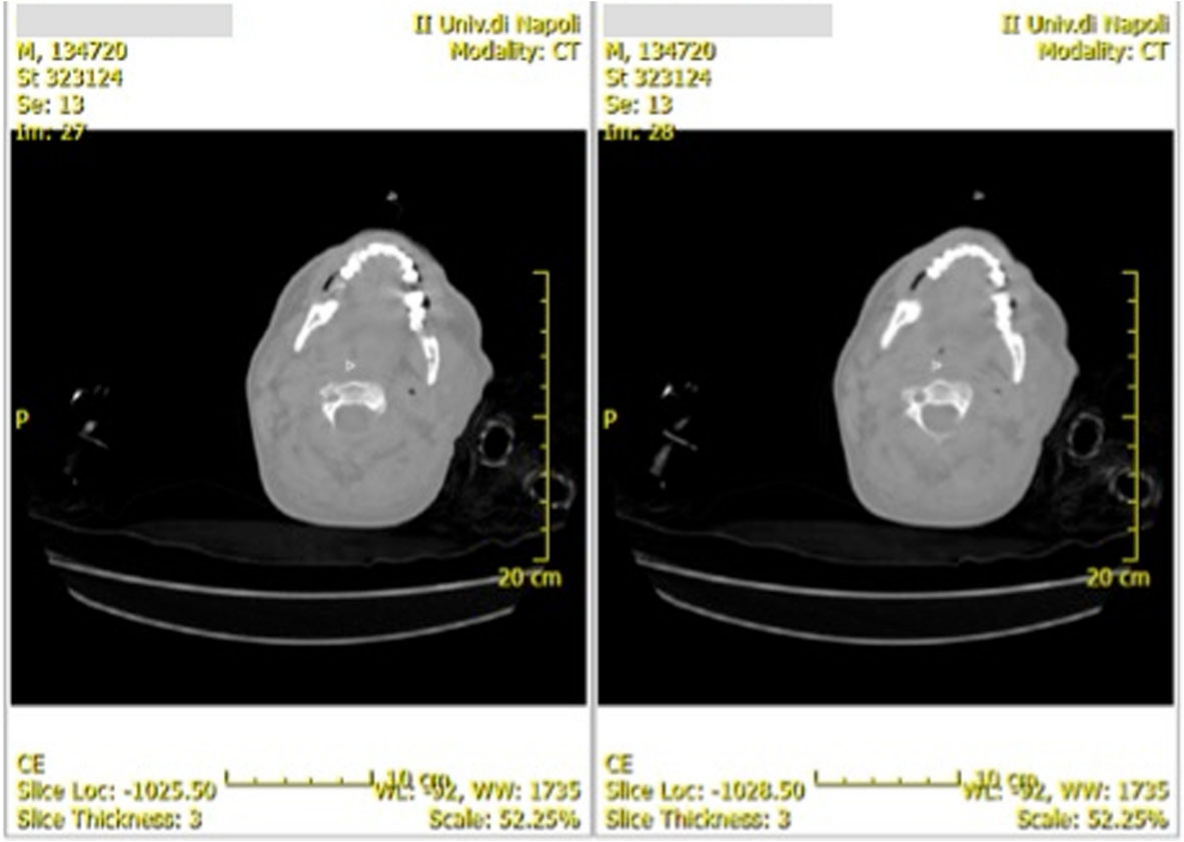
Thoracic computed tomography scan

All his skin was pallid except for the left side of his neck. He had a large warm mass on the left side of his neck, which extended from his mouth to his left supraclavicular region. At thoracic auscultation there were no lung sounds at the left side and some wheezes at the right side. He also presented peripheral edema. A neurological examination was not done because he was deeply sedated (Ramsay Sedation Scale 6 and Glasgow Coma Scale 3). Chest and neck computed tomography with contrast medium showed: a wide abscess in left parotid-masseter region that extended from the floor of his mouth up to the ipsilateral inferior parapharyngeal compartment, this lesion appeared to be liquefied with areas of air pockets; severe bilateral pleural effusion; and an abscess in his anterior mediastinum that extended from median to left paramedian area (Figs. 1 and 2). He immediately underwent bilateral thoracotomy and left cervicotomy with abscess drainage and left superior and inferior third molars (2.8 and 3.8 tooth), whose dental roots were necrotic, and then he was admitted to our ICU because of septic shock: qSequential Organ Failure Assessment (qSOFA) 3; SOFA score 12. All cultural examinations were done (chest drain samples, blood cultures, bronchial aspirate culture, and urine sample); in particular, the blood sample culture (VersaTREK REDOX® 1 and 2) revealed the presence of methicillin-resistant *Enterococcus raffinosus*, CRAB, and CR-Kp. In addition, the culture from the abscess drainage also revealed CRAB and CR-Kp. The cultures were identified with standard methods (we did not have rapid test) and minimal inhibitory concentration (MIC) susceptibility test. The stains of pus collected were both Gram positive and Gram negative. The blood samples also revealed: white blood cells (WBC) 17,500/mL (neutrophils 85%, lymphocytes 8%), red blood cell (RBC) 2.79 × 10^6^/ml, hemoglobin 7.2 g/dl, hematocrit 22.8%, platelet 118 × 10^3^/ml, C-reactive protein (CRP) 12.3 mg/L (normal range 0–10 mg/L), PCT 12 ng/mL (normal 0.15 ng/mL), endotoxin activity assay (EAA) 0.72 (EAA in the low range < 0.4) with a negative predictive value of 95.1% from risk of culture-proven Gram-negative infection, aspartate transaminase 21 U/L, alanine transaminase 24 U/L, total bilirubin 0.46 mg/dl, unconjugated bilirubin 0.11 mg/dl, conjugated bilirubin 0.35 mg/dl, serum creatinine 1.4 mg/dl, urea 53 mg/dl, and body temperature 40 °C. Hemodynamic parameters monitored with Vigileo (specific monitor that analyzes arterial blood pressure waveform and its changes) were: cardiac output (CO) 2.1 L/minute (normal range 4.0–8.0 L/minute), systemic vascular resistance (SVR) 400 dynes · second/cm^5^ (normal range 800–1200 dynes · second/cm^5^), MAP 50 mmHg (with dobutamine 8 μg/kg per minute and norepinephrine 0.3 μg/kg per minute), and urinary output > 0.5 mL/kg per hour with Acute Kidney Injury (AKI) scale 1. The blood gases showed PaO_2_/FiO_2_ 171 and lactate 2.5 mMol/L (normal range up to 1.9 mMol/L). He started an antibiotic therapy with: linezolid 1200 mg/day, colistin 9,000,000 UI/day, rifampicin 600 mg/day, and tigecycline 100 mg/day. At 36 hours after the surgical and antibiotics therapy we did not notice a significant improvement so we decided to start a combined therapy with 250 ml/kg per day IgM-enriched immunoglobulin preparation for 3 consecutive days, together with direct hemoperfusion therapy with immobilized polymyxin B cartridges for 2 hours a day for 2 consecutive days (blood flow 100 ml/minute). Toraymyxin (PMX 20-R; Toray Industries, Tokyo, Japan) is a selective blood endotoxin removal cartridge. PMX 20-R is a cartridge composed of polymyxin B covalently bonded to polystyrene-derivative fibers. Blood flow direction is well controlled by adopting a radial flow system. PMX 20-R treatment occurs by hemoperfusion at a blood flow rate of approximately 80–120 mL/minute. Heparin is preferably used as an anticoagulant. Pentaglobin (IgM-enriched immunoglobulin; Biotest) is a plasma-derived solution with the sequent composition: 12% IgM – 76% IgG – 12% IgA. Several mechanisms of action have been postulated for Pentaglobin: direct activity of antibodies, neutralization of endotoxin, enhanced clearance of lipopolysaccharide (LPS), and reduction of classical complement pathway. Approximately 3 days after the beginning of this multimodal intensive and progressive treatment, gradual improvements in hemodynamics (MAP 85 mmHg without norepinephrine, CO 5.2 L/minute, urinary output > 0.5 ml/kg per hour), blood gases, and inflammatory markers (CRP 2.3 mg/dl, PCT 1 ng/ml, EAA < 0.6, body temperature 36.5 °C, and lactate 0.3 mMol/L) were achieved (Table 1). We noted a fluid overload of our patient during the first 36 hours before starting the therapy with Toraymyxin and Pentaglobin but there was a rapid and immediate recovery of a normal urine output after the beginning of that therapy, following on from increasing MAP.

**Table 1:** Hemodynamic and septic parameters

	T0	T1	T2
CRP (mg/dl)	12.3	12	2.3
PCT (ng/ml)	3.2	3	1
EAA	0.71	0.6	0.5
FEVER (°C)	40	38	36.5
SVR (dynes second/cm^5^)	400	600	1200
CO	2.1	3.0	5.2
MAP	50	70	85

T0: Before the beginning of IgM-enriched immunoglobulin preparation (Pentaglobin) and direct hemoperfusion therapy with polymyxin B immobilized fiber cartridges

T1: 24 hours after the beginning of IgM-enriched immunoglobulin preparation (Pentaglobin) and direct hemoperfusion therapy with polymyxin B immobilized fiber cartridge

T2: 72 hours after the beginning of IgM-enriched immunoglobulin preparation (Pentaglobin) and direct hemoperfusion therapy with polymyxin B immobilized fiber cartridge

*CO* cardiac output, *CRP* C-reactive protein, *EAA* endotoxin activity assay, *MAP* mean arterial pressure, *PCT* procalcitonin, *SVR* systemic vascular resistance

**Fig. 3:**
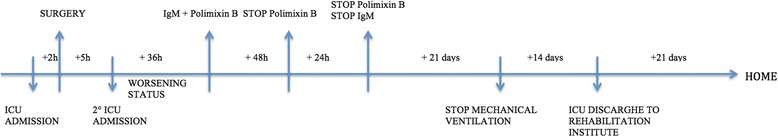
Case report timeline. ICU intensive care unit

So the weaning from mechanical ventilation started. Approximately 3 weeks after his admission to ICU, he was successfully weaned from mechanical ventilation. His ICU course was complicated also by polyneuropathy, myopathy, and hyperthyroidism. Finally, after 5 weeks, he was transferred to a rehabilitation institute. He was discharged home 3 weeks later (Fig. 3). At 6 months follow-up he was discharged to home without tracheostomy and was starting to work again.

## Discussion

In this case report we describe a rare case of DNM due to Gram-negative and Gram-positive multidrug-resistant bacteria. We obtained the resolution of DNM by assembling conventional surgical therapy and a novel treatment with IgM-enriched immunoglobulin preparation and direct hemoperfusion therapy with immobilized polymyxin B cartridges, even though there was no recommendation for this treatment in this case and there was a lack of literature.

AM is a severe, life-threatening infection of the mediastinal connective tissues, interpleural spaces, and surrounding thoracic organs. DNM is a rare complication of odontogenic infection [8] arising generally from the second or third molar. Other less common infections include acute tonsillitis, and retropharyngeal and peritonsillar abscess. This abscess can rupture into the submandibular and parapharyngeal spaces and reach the mediastinum mainly along the retropharyngeal space or along the perivascular and pretracheal spaces [9, 10]. The effect of gravity and the negative intrathoracic and pleural pressure during inspiration, and the absence of barriers in the facial planes are important pathophysiological factors in the extension of deep neck infections of the mediastinum [10]. In a systematic review published in April 2016 by Prado-Callero *et al*., the authors analyzed 26 studies with a total of 480 patients. DNM was reported to be limited to the upper mediastinum in 189 patients (39%) and extended to the inferior mediastinum in 249 patients (51%) [1]. The origin of DNM was pharyngeal (acute tonsillitis, retropharyngeal and peritonsillar abscess) in 204 patients (45%), odontogenic abscess in 163 patients (36%), from other causes in 83 patients (18%), and not reported in 30 patients [1].

In addition the presence of coexisting morbidities, such as diabetes mellitus (DM), alcoholism, tobacco smoking, chronic renal failure, and liver cirrhosis, can facilitate this rapid extension and increase the occurrence of complications including bilateral empyema, purulent pericarditis, AKI requiring hemodialysis, prolonged mechanical ventilation, and septic shock. Endo *et al*. [11] classified DNM according to the anatomic extent: type I infection above the carina (localized form); and type II infection below the tracheal bifurcation (diffuse form), which is subdivided into type IIA (lower anterior mediastinum) and type IIB (lower posterior mediastinum).

The mortality under current “standard treatment” is reported to be between 20 and 40% [12, 13] mostly as a consequence of multiorgan failure (MOF).

The etiological organisms of DNM are mostly mixed, aerobic and anaerobic [5, 14]. Although the main microorganisms implicated are Gram-positive bacteria (e.g. staphylococci), Gram-negative pathogens can also be a relevant cause of DNM [15].

A European network of national surveillance systems on antimicrobial resistance (EARS-Net), coordinated and financed by European Centre for Disease Prevention and Control (ECDC), has been created to collect data from 29 European countries to analyze temporal and spatial trends of the phenomenon. European data confirm an increase in resistance to third-generation cephalosporins, fluoroquinolones, and aminoglycosides especially in *Escherichia coli* and in *K. pneumoniae*, responsible for urinary tract infections, sepsis, and other health care-related infections. These resistances are often combined, generating multi-resistant bacteria. In recent years, resistance to carbapenem has appeared, making some infections untreatable. Data collected during the Antibiotic Resistance-Istituto Superiore di Sanità (AR-ISS) project have confirmed that Italy is among the countries with the highest levels of resistance in most pathogenic species under surveillance, namely 32.9% of *K. pneumoniae* isolates were resistant to carbapenem, 33.6% of *Staphylococcus aureus to methicillin*, and 28.7% and 43.9% of *E. coli* isolates to third-generation cephalosporins and fluoroquinolones, respectively [16].

Compared with other microorganisms, DNM associated with Gram-negative pathogens has higher rates of in-hospital death [16]. Invasive infections associated with carbapenem-resistant Enterobacteriaceae (CRE) pose a serious challenge to clinicians. CRAB has increasingly emerged as an important nosocomial pathogen [17, 18], and the postoperative mediastinitis caused by CRAB is rare. It has severe complications associated with high morbidity and mortality. Treatments with polymyxin (polymyxin b or colistin), carbapenem, or their combinations are basically supported by observational studies [19, 20].

The significance of interplay between immune system-related substances and bacterial toxins in the pathogenesis of sepsis and the subsequent deleterious effects on organ function is well known. Apparently, endotoxins/LPS and lipoteichoic acid are the key toxins produced by Gram-negative and Gram-positive bacteria, respectively, playing an important role in inducing a systemic inflammatory response [21, 22] and it was found that removing them from circulation can have beneficial effects. Polymyxin B is a well-known antibiotic with high affinity for endotoxin and it is able to neutralize it, although it is associated with neurotoxicity and nephrotoxicity, which precludes its systemic use. In 1994, the Japanese company Toray (Tokyo, Japan) designed Toraymyxin (PMX-20R device) in order to adsorb and neutralize endotoxins from the circulation by using polymyxin B in extracorporeal modality, fixing it covalently on polystyrene fiber matrix. It has been shown in meta-analyses and many experimental studies [24, 25] that extracorporeal endotoxin adsorption using this method is beneficial in the management of patients with sepsis [23], especially in managing the septic shock and in the reversal of organ dysfunction. Some researchers [24, 25] reported that selective removal of endotoxins/LPS can specifically improve hemodynamic and respiratory functions, and this was explored in a systematic review by Cruz *et al*. [26] concluding that it is associated with an improved MAP, inotrope use, levels of PaO_2_/FiO_2_ ratio, and decreased mortality.

Several studies reported improvement in SVR [27–31], CO, or cardiac index [29, 31]. However, pooled data seem to indicate that polymyxin B hemoperfusion should be able to increase blood pressure with the reduction of vasoactive agents [29, 32–35] compared with conventional treatments [36, 37]. Polymyxin B hemoperfusion might improve the oxygenation index and oxygenating lung functions [33, 34] possibly because of a reduction in pulmonary epithelium injury, permeability, and endothelial damage. Polymyxin B hemoperfusion improves gas exchange (PaO_2_/FiO_2_ ratio), but there was only a single randomized controlled trial (RCT) that reported this outcome and it showed only a non-significant positive trend [37]. After polymyxin B hemoperfusion, reduced levels of other mediators, such as interleukin (IL)-6 [38, 39], IL-10 [38, 39], IL-18 [40], tumor necrosis factor (TNF)-α [39, 41], metalloproteinase-9 [27], plasminogen activator inhibitor-1 [39, 41], neutrophil elastase [42, 43], platelet factor-4 [40], β-thromboglobulin [40], soluble P selectin [40], and endogenous cannabinoids, were also observed [44].

However, there are currently no recommendations supporting (nor against) the routine use of blood purification techniques in sepsis in International Guidelines for Management of Sepsis and Septic Shock [45]. Moreover, a recent meta-analysis demonstrated a favorable effect on overall mortality with this technique [46]. However, these findings must be interpreted with caution because of the suboptimal quality of studies. The other treatment used in these patients was IgM-enriched polyclonal immunoglobulin (IVIgGM). Immunoglobulins constitute an innovative product with a wide range of clinical use.

In the 2016 Surviving Sepsis Campaign, there is a weak recommendation with low quality of evidence concerning the use of immunoglobulins [45]. One larger multicenter RCT [47] in adult patients found no benefit for intravenously administered immunoglobulin (IVIg). The Cochrane meta-analysis [48] differentiates between standard polyclonal intravenously administered immunoglobulins (IVIgG) and IVIgGM. In ten studies with IVIgG, mortality between 28 and 180 days was 29.6% in the IVIgG group and 36.5% in the placebo-group, and for the seven studies with IVIgGM, mortality between 28 and 60 days was 24.7% in the IVIgGM group and 37.5% in the placebo-group. The certainty of the studies was rated as low for the IVIgG trials, based on the risk of bias and heterogeneity, and as moderate for the IVIgGM trials, based on risk of bias. However, the recent Cochrane analysis revealed no survival benefits. These findings are similar to those of an older meta-analysis [49] from other Cochrane authors. One systematic review [50] showed a reduction in death with immunoglobulin treatment; however, the results of only high quality trials did not show a statistically significant difference. Finally, Laupland *et al*. [49] found a significant reduction in mortality with the use of IVIg treatment.

Recently, two meta-analyses, using less strict criteria to identify sources of bias found significant improvement in patient mortality with IVIg treatment [51, 52]. Finally, there are no cut-offs for plasma IVIg levels in patients with sepsis, for which substitution with IVIg improves outcome data [52]. Subgroup effects between IgM-enriched and non-enriched formulations reveal significant heterogeneity. The low certainty of evidence led to the grading as a weak recommendation. The statistical information that comes from the high quality trials does not support a beneficial effect of polyclonal IVIg.

## Conclusions

In our case we decided to start the treatment with immunoglobulins and polymyxin B hemoperfusion after the failure of conventional surgical and medical therapy and in light of elevated endotoxin level. This case of DNM was difficult to treat because of the presence of multidrug-resistant bacteria probably related to the delay in diagnosis and the use of first-line antibiotics for treatment of incorrectly assumed pneumonia. After the surgical source control, only with the combined use of IgM-enriched immunoglobulin preparation and polymyxin B hemoperfusion as integration antibiotic therapy, did we obtain an improvement of hemodynamic and respiratory parameters. CO, cardiac index, SVRs, central venous saturation of oxygen (ScVO_2_), and oxygen delivery (DO2I) improved. PaO_2_/FiO_2_ ratio improved, endotoxemia and PCT decreased, and vasoactive agents were interrupted. Despite the contrasting data on the use of immunoglobulins and polymyxin B hemoperfusion in septic shock and AM caused by either Gram-negative or Gram-positive multidrug-resistant bacteria, we obtained an improvement of clinical conditions and the survival of our patient against all odds.
